# Environmental sampling for SARS-CoV-2 at a reference laboratory and provincial hospital in central Viet Nam, 2020

**DOI:** 10.5365/wpsar.2020.11.4.002

**Published:** 2021-07-12

**Authors:** Thái Hùng Đỗ, Văn Thành Nguyễn, Thế Hùng Đinh, Xuân Huy Lê, Quang Chiêu Nguyễn, Văn Quân Lê, Bảo Triệu Nguyễn, Ngọc Bích Ngân Nguyễn, Thị Ngọc Phúc Nguyễn, Kim Mai Huỳnh, Hoàng Long Trịnh, Thị Kim Trang Lê, Thùy Dung Diệp, Thủy Thị Thu Đỗ, Hiền Thị Thu Bùi, Alyssa M Finlay, Quốc Việt Nguyễn, Philip L Gould

**Affiliations:** a Pasteur Institute of Nha Trang, Nha Trang, Viet Nam.; b Binh Thuan General Hospital, Binh Thuan, Viet Nam.; c Binh Thuan Center for Disease Control, Binh Thuan, Viet Nam.; d United States Centers for Disease Control and Prevention, Hanoi, Viet Nam.; e Provincial Department of Health, Binh Thuan, Viet Nam.

## Abstract

**Objective:**

To determine whether environmental surface contamination with severe acute respiratory syndrome coronavirus 2 (SARS-CoV-2) occurred at a provincial hospital in Viet Nam that admitted patients with novel coronavirus disease 2019 (COVID-19) and at the regional reference laboratory responsible for confirmatory testing for SARS-CoV-2 in 2020.

**Methods:**

Environmental samples were collected from patient and staff areas at the hospital and various operational and staff areas at the laboratory. Specimens from frequently touched surfaces in all rooms were collected using a moistened swab rubbed over a 25 cm^2^ area for each surface. The swabs were immediately transported to the laboratory for testing by real-time reverse transcription polymerase chain reaction (RT–PCR). Throat specimens were collected from staff at both locations and were also tested for SARS-CoV-2 using real-time RT–PCR.

**Results:**

During the sampling period, the laboratory tested 6607 respiratory specimens for SARS-CoV-2 from patients within the region, and the hospital admitted 9 COVID-19 cases. Regular cleaning was conducted at both sites in accordance with infection prevention and control (IPC) practices. All 750 environmental samples (300 laboratory and 450 hospital) and 30 staff specimens were negative for SARS-CoV-2.

**Discussion:**

IPC measures at the facilities may have contributed to the negative results from the environmental samples. Other possible explanations include sampling late in a patient’s hospital stay when virus load was lower, having insufficient contact time with a surface or using insufficiently moist collection swabs. Further environmental sampling studies of SARS-CoV-2 should consider including testing for the environmental presence of viruses within laboratory settings, targeting the collection of samples to early in the course of a patient’s illness and including sampling of confirmed positive control surfaces, while maintaining appropriate biosafety measures.

Viet Nam recorded its first two cases of infection with severe acute respiratory syndrome coronavirus 2 (SARS-CoV-2), the virus that causes novel coronavirus disease 2019 (COVID-19), on 23 January 2020. (*1*) As of 12 May 2021, the country had recorded 3658 COVID-19 cases; 2636 (72.0%) people had recovered, 35 (1.0%) deaths were reported and 983 (26.9%) cases were still under observation. (*2*)

The Serology and Cell Culture Laboratory at the Pasteur Institute of Nha Trang is a Biosafety Level 2 facility that serves as the reference laboratory for 11 provinces in the Central Coast region of Viet Nam(**Fig. 1**). Between 9 March and 9 April 2020, the Pasteur Institute tested 6607 patient respiratory specimens (oral and nasopharyngeal swabs) for SARS-CoV-2 using real-time reverse transcription polymerase chain reaction (RT–PCR). Testing was conducted on repeated specimens from confirmed cases to monitor viral RNA shedding and to inform medical management and disposition. During this time, 15 COVID-19 cases were detected within the area serviced by the Pasteur Institute.

Binh Thuan General Hospital, located in one of the southern provinces in the Central Coast region (**Fig. 1**), cared for 9 of the 15 COVID-19 cases from the Central Coast region (60%) from 9 March to 9 April 2020. The 9 cases were epidemiologically linked as one cluster, either as household contacts or close contacts at work. Only the index case was symptomatic, with onset on 5 March and symptoms including fever, productive cough and sore throat; the index case was hospitalized on 9 March after a respiratory specimen tested positive for SARS-CoV-2 by real-time RT–PCR. The other 8 cases were identified through contact tracing from the index case or were subsequently identified positive contacts, and all were asymptomatic. All 9 cases were admitted to the general hospital between 9 and 11 March, due to Viet Nam’s policy of isolating positive cases within hospitals even when they are asymptomatic. The policy is a more aggressive isolation approach than that in many countries but contributes to the relative success in controlling the ongoing pandemic. (*3*) The 9 patients were admitted to 3 separate rooms and had periodic testing of respiratory specimens for SARS-CoV-2 to monitor for illness and the clinical course.

**Figure 1:**
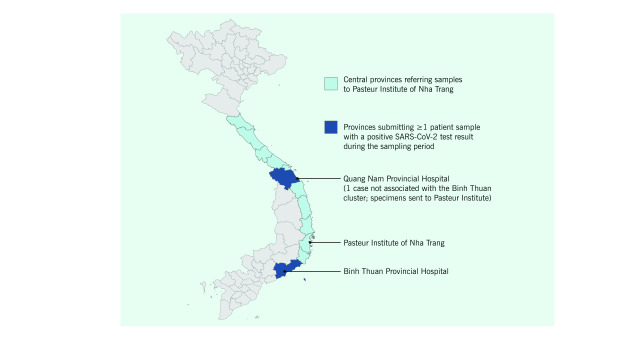
Map of the Central Coast region of Viet Nam, showing the location of Binh Thuan General Hospital and the Pasteur Institute of Nha Trang and the areas covered by the sampling for SARS-CoV-2, 2020

[**insert** Figure 1]

During the time the cases were hospitalized, there were other patients with other medical problems housed within different departments of the general hospital.

The SARS-CoV-2 virus has been shown to persist on a variety of surfaces, (*4*, *5*) with reports of environmental contamination in patient care settings. (*6*-*8*) However, understanding is limited about environmental contamination in laboratories handling patient specimens. The objective of this study was to assess whether any environmental contamination occurred at the general hospital or the laboratory at the Pasteur Institute at the time when COVID-19 cases were hospitalized post-diagnosis.

## Methods

### Sample collection

Staff at both Binh Thuan General Hospital and the reference laboratory at the Pasteur Institute of Nha Trang were trained virtually in sample collection through lectures, demonstrations and a question and answer session. A rapid practical competency assessment was performed by observing staff conducting procedures via real-time video conferencing. At the general hospital, staff collected environmental samples during 6 days, between 26 and 31 March, from all 3 rooms (each with a separate bathroom) used to isolate COVID-19 cases, 2 staff rooms used by health care workers caring for COVID-19 cases and one control room where internal medicine patients were treated who had no signs and symptoms of or known epidemiological risk factors for COVID-19, in accordance with World Health Organization (WHO) guidelines. (*9*) Frequently touched surfaces, including those touched by the COVID-19 cases (e.g. light switches, doorknobs and bed rails; **Table 1**), were sampled before and at least 1 hour after afternoon cleaning.

**Table 1:** Locations where environmental surfaces were sampled for SARS-CoV-2 and number of samples collected from each surface at Binh Thuan General Hospital, Viet Nam, 26–31 March 2020

Surface sampled	Room and number of samples^a^			
	COVID-19 patients	Control patients	Administration	Staff breakroom
Doorknob	36	2	-	12
Bedside rails	36	2	-	-
Call button	36	2	-	-
N95 respirator (of technician or nurse; sampled before and after use)	36	2	-	-
Bedside daily medical record	36	2	-	-
Air vent	36	2	-	-
Bathroom doorknob	36	2	-	-
Bathroom faucet handles	36	2	-	12
Sink	36	2	-	-
Computer keyboard	-	-	12	-
Chart cover	-	-	12	-
Telephone	-	-	12	-
Staff clothing	-	-	-	12
Tabletop	-	-	-	12
Light switch	-	-	-	12
Staff mobile phone	-	-	-	12
**Total samples collected ** **(*n* = 450)**	**324**	**18**	**36**	**72**

^a^ Empty cells indicate that no samples were collected from that particular surface.

At the Pasteur Institute, microbiology laboratory staff collected environmental samples from 6 rooms: those used for receiving, processing and extracting specimens, and preparing the master mix for RT–PCR; as well as the PCR machine room; and the staff room. Frequently touched surfaces (e.g. doorknobs, countertops, light switches and faucet handles; **Table 2**) likely to be touched by laboratory workers processing patients’ specimens were swabbed on 6 days between 23 March and 9 April. Two samples were collected each day: 1 hour before and 1 hour after afternoon cleaning and decontamination.

**Table 2:** Locations where environmental surfaces were sampled for SARS-CoV-2 and number of samples collected from each surface in the Serology and Cell Culture Laboratory at the Pasteur Institute of Nha Trang, Viet Nam, 23 March–9 April 2020

Surface sampled	Room and number of samples^a^					
	Specimen receiving	Specimen processing	Specimen extracting	Master mix room	PCR machine room	Staff breakroom
Doorknob	12	12	12	12	-	-
Biosafety cabinet floor	12	12	12	-	-	-
Specimen testing request form	12	-	-	-	-	-
Tabletop where specimens received	12	-	-	-	-	-
Light switch	12	12	12	-	-	-
Faucet handles	12	12	12	-	-	-
Sample transfer pipette	-	12	12	-	-	-
PPE changing location floor	-	12	-	-	-	-
Centrifuge	-	12	-	-	-	-
Staff members’ blouses	-	-	12	-	-	-
PCR platform	-	-	-	12	-	-
Computer keyboard and mouse	-	-	-	-	12	12
Buttons on RT–PCR machine	-	-	-	-	12	-
Test result form	-	-	-	-	-	12
**Total (*n* = 300)**	**72**	**84**	**72**	**24**	**24**	**24**

PPE: personal protective equipment; RT–PCR: real-time reverse transcription polymerase chain reaction.

^a^ Empty cells indicate that no samples were collected from that particular surface.

Staff working in the hospital and the laboratory had throat specimens collected and tested by real-time RT–PCR at the Pasteur Institute before the environmental samples were collected in either location.

Surfaces were sampled using Puritan standard sterile polyester tipped applicators with a solid polystyrene handle (number 25–806 1PD; Puritan Medical Products, Guilford, Maine, USA). Following WHO’s environmental sampling protocol, (*9*) for each sample, a single swab dipped into a universal transport medium tube (UTM 330C transport system 16 × 100 mm tube with 3 mL UTM medium, COPAN Diagnostics, Murrieta, California, USA) was used to swab a 25 cm^2^ surface area and was immediately returned to the tube. Specimens were either transported immediately or stored at 2–8 °C while awaiting transport to the laboratory and shipped to arrive at the laboratory within 72 hours from collection; at the laboratory, they were processed immediately or stored at –70 °C.

### Laboratory testing

All environmental samples, including from the patients’ room used as a control, were tested for SARS-CoV-2 by real-time RT–PCR using the Charité Berlin Research Institute protocol, including using positive and negative controls. (*10*) The RNA was extracted manually by using the QIAamp DSP viral RNA mini extraction kit (QIAGEN, Venlo, the Netherlands) according to the manufacturer’s specifications. The positive controls were prepared by Viet Nam’s National Institute for Hygiene and Epidemiology from cultured virus at 10^−3^ dilution derived from patients’ specimens and shared with the laboratory at the Pasteur Institute; cycle threshold (CT) values for the positive control were between 26 and 28 cycles.

### Cleaning practices

Information on the cleaning regimens at the Pasteur Institute and the general hospital were obtained by the staff at each institution.

## Results

### Testing conducted at the Pasteur Institute

**Figure 2:**
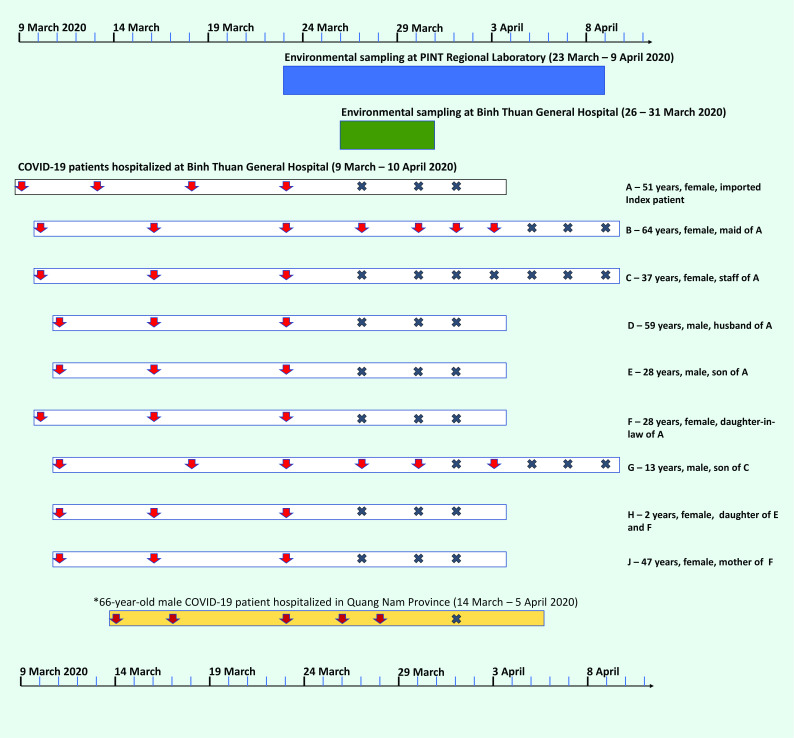
Timeline of hospitalization of COVID-19 patients at the Binh Thuan General Hospital and sampling of environmental surfaces at the Pasteur Institute of Nha Trang (23 March–9 April 2020) and the general hospital (26–31 March 2020). Red arrows indicate patients’ positive SARS-CoV-2 specimens by date of collection and a grey X indicates a negative specimen. *Patient was an imported case not connected to the Binh Thuan cluster

During the environmental sampling period at the Pasteur Institute’s laboratory (23 March to 9 April 2020), the workload comprised testing for SARS-CoV-2 by real-time RT–PCR of 6607 respiratory specimens from patients. Of these, 19 (0.3%) specimens were positive for SARS-CoV-2 (**Fig. 2**).

[**insert** Figure 2]

From patients A–H and J on 23 March (9 positive tests);
From patient B on 27 and 30 March and on 1 and 3 April (4 positive tests);
From patient G on 27 and 30 March and on 3 April (3 positive tests);
From a patient in Quang Nam (not part of the cluster in Binh Thuan, but who had tests submitted to the Pasteur Institute laboratory) on 23, 26 and 28 March (3 positive tests).

Two patients admitted to the general hospital had respiratory specimens that were positive for SARS-CoV-2 during the hospital’s environmental sampling period of 26–31 March 2020 (patients B and G). The CT values in the specimens positive for the E gene ranged from 20.00 to 31.57, with an average of 27.25; for the *RdRp* gene, the CT values ranged from 23.72 to 37.10, with an average of 31.30.

### Infection prevention and control practices

The cleaning regimen at the general hospital included twice daily cleaning of surfaces – including medical equipment, beds, dining tables, television controls, call buttons, doors, bedside cupboards and bed rails – with a disinfectant solution containing 0.05% chlorine. Cleaners worked in sequence from low-risk areas to high-risk areas. Floors were also cleaned twice a day with the same disinfectant solution.

At the Pasteur Institute twice weekly cleaning of surfaces, floors and doors was done with 0.05% chlorine solution. Daily cleaning and disinfection of other surfaces (e.g. desks, biosafety cabinet floors, pipettes, doorknobs) was done with alcohol (70%) at the end of the day or when spills occurred. Ultraviolet light was used for 15 minutes at the beginning and end of the workday to disinfect the laboratory.

Other infection prevention and control (IPC) measures at the Pasteur Institute included 24-hour room ventilation, with temperature and humidity checked daily. During the survey period, the average temperature was 23 ± 2 °C and humidity was 62 ± 5%. There are also exhaust fans to the outdoors and certified biosafety cabinets used to avoid potential aerosol and droplet exposure and these are either exhausted through a high-efficiency particulate air (HEPA) filter (where samples are received) or to the outdoors (where samples are processed). While handling potentially infectious specimens, laboratory workers wear suits with hoods, eye protection, N95 respirators and gloves.

### Environmental samples

A total of 750 environmental specimens were collected (**Tables 1** and **2**). At the Pasteur Institute, 300 environmental samples were collected from 6 rooms (**Table 2**):

72 from the specimen receiving room – 12 each (1 sample 2 times per day for 6 days) from the biosafety cabinet floor, patient request forms, specimen receiving table, light switch, faucet handles and doorknob;
84 from the specimen processing room – 12 each (1 sample 2 times per day for 6 days) from the biosafety cabinet floor, light switches, doorknobs, faucet handles, sample transfer pipettes, centrifuge and floor of the changing station for personal protective equipment (PPE);
72 from the extraction room – 12 each (1 sample 2 times per day for 6 days) from the light switches, doorknobs, faucet handles, biosafety cabinet floors, sample transfer pipettes and staff members’ blouses;
24 from the solution room – 12 each (1 sample 2 times per day for 6 days) from the PCR platform and doorknobs;
24 from the PCR machine room – 12 each (1 sample 2 times per day for 6 days) from the computer mouse, computer keyboard and touchpad of the RT–PCR machine;
24 from the staff breakroom – 12 each (1 sample 2 times per day for 6 days) from the computer mouse and keyboards and from test result forms;

All 300 samples collected from the Pasteur Institute were negative for SARS-CoV-2.

At the general hospital, 450 samples were collected from 6 rooms (**Table 1**):

324 from the 3 rooms where COVID-19 patients were isolating – 12 samples (2 each day, 1 hour before and 1 hour after the afternoon cleaning for 6 days) from frequently touched sites including the doorknob, bed rails, call buttons, bedside daily medical records, air vents, private bathroom doorknobs and faucet handles, plus from the N95 respirator of the technician or nurse used in each room, sampled before and after use;
18 from the room with the control patients – 2 samples from the same 9 locations as the patients’ rooms;
36 from an administrative room – 2 samples per day from 3 locations: keyboard, chart cover and telephone;
72 from a staff breakroom – 12 samples (2 each day, 1 hour before and 1 hour after the afternoon cleaning for 6 days) from the doorknob, faucet handles, tabletop, light switch, staff clothing and mobile phones.

All 450 samples from the general hospital were negative for SARS-CoV-2.

There were 20 health care workers at the general hospital and 10 laboratory workers at the Pasteur Institute involved in the study or in caring for patients who tested positive for SARS-CoV-2 by real-time RT–PCR; all 30 staff were tested at the start of the study period and were negative for SARS-CoV-2 by real-time RT–PCR.

## Discussion

All environmental samples collected from a hospital and reference laboratory setting in the Central Coast of Viet Nam that cared for and provided services for COVID-19 patients were negative for SARS-CoV-2. The samples from the hospital room with control patients were included with an expectation that some of the environmental samples from other patients’ rooms might be positive, although this proved not to be the case in this investigation. Specimens collected from staff members in both settings were also negative for SARS-CoV-2. Surface samples comprised multiple, frequently touched locations within the hospital and the laboratory throughout the life cycle of the specimens, as well as other areas frequented by staff. To our knowledge, this investigation is the first to document environmental sampling for SARS-CoV-2 within a laboratory setting.

This investigation did not provide any evidence of surface contamination occurring within either the reference laboratory that provides SARS-CoV-2 testing for 11 provinces in Viet Nam’s Central Coast or within a provincial hospital caring for a cluster of COVID-19 patients. The facilities’ IPC measures may have contributed to these findings. The laboratory was mechanically ventilated with fresh-air supply and exhaust fans and used certified biosafety cabinets to control potential aerosol and droplet exposure. Staff also followed strict laboratory biosafety protocols. The hospital reported adherence to environmental cleaning and disinfection regimens using 0.05% chlorine solution twice daily, in accordance with national and WHO guidelines, (*11*) all with the purpose of limiting surface contamination. The negative SARS-CoV-2 test results from routine screening of workers at both the hospital and laboratory during the study period further support the effectiveness of IPC measures and the lack of health care–associated transmission.

Another possible explanation for the lack of positive environmental samples from the hospital is that cases were no longer shedding virus at the time the environmental samples were collected. Sampling occurred late in the patients’ clinical course, at 15–17 days after hospital admission following the index case’s positive specimen. Sample collection was delayed while approvals to conduct the study were obtained. Culturable virus is often absent from patients who have mild to moderate illness at days 8–9 post-symptom onset (*7*) and low in asymptomatic patients. (*7*, *12*-*14*) Peak viral shedding occurs early, at around 4–6 days post-infection or a few days before and after onset of symptoms (when symptomatic), (*7*, *13*-*15*) so the delay in sampling limits the interpretation of the quality of IPC practices.

All but one of the COVID-19 cases in this study were asymptomatic and, therefore, potentially had less viral shedding, making it less likely that positive environmental samples would be obtained. A recent study showed that the presence and concentration of environmental contamination with SARS-CoV-2 in patients’ rooms and air vents within those patients’ rooms were associated with patients being early in the course of their illness (having symptoms for < 1 week), when viral loads are known to peak. (*7*) Also, viral remnants may have been rapidly degraded in the environment.

Additionally, as contamination is typically not uniformly distributed on surfaces, the sampling might have missed potential evidence of virus in areas not sampled, or the contamination might have been below the limit of detection. Given the use of a single swab per surface, it is possible that the swabs may not have remained sufficiently moist or that the contact time during collection may have been insufficient. The study design also did not include an experimentally contaminated surface to use as a known positive control while maintaining appropriate biosafety measures (e.g. using denatured virus). All of these may have contributed to the negative findings in this study. Even so, virtual training was conducted for the staff who performed the sampling, and surface sampling technique was also observed in real time using a mobile phone with video access. Recent environmental sampling of very-high-touch surfaces in public transportation venues at the height of the pandemic in Italy have similarly returned negative results. (*16*)

In conclusion, our study found no environmental contamination by SARS-CoV-2 among 750 samples taken from a hospital treating COVID-19 patients and a reference laboratory conducting testing for 11 provinces in Viet Nam. The facilities’ IPC measures may have contributed to these results, although other possible explanations include sampling late in the patient’s hospital stay, using insufficient contact time to collect samples or using insufficiently moist swabs. Further environmental sampling studies of SARS-CoV-2 should consider including testing for the environmental presence of viruses within the laboratory setting and consider including additional quality assurance methods, such as a positive control surface, while ensuring appropriate biosafety measures. These studies should also strive to collect specimens as early as possible in each case’s infection to minimize potential loss due to reductions in viral load over time.

### 

#### Disclaimer

The findings and conclusions in this report are those of the authors and do not necessarily represent the views of the United States Centers for Disease Control and Prevention or the Ministry of Health of Viet Nam.
